# The Paddington Green Hospital for Children

**Published:** 1911-06-03

**Authors:** 


### HOSPITALS OF TO-DAY.

### VII.—THE PADDINGTON GREEN HOSPITAL FOR CHILDREN.

To-day this hospital will be closed until October. The extension of the out-patient department, which is now proceeding, makes this a necessary step, and the moment becomes a happy one to summarise the hospital's achievement and to state as shortly as possible the present position of affairs.

The Children's Hospital on Paddington Green is one of the comparatively few institutions which have not let the years slide by with no apter record than a series of annual reports, and Mr. W. H. Pearce, the Secretary, has earned the thanks of posterity for the historical leaflet his devotion has compiled. This leaflet contains an interesting example of the potentiality for good of small beginnings, and we are glad to quote the following passage: "In 1862 the 'North-West London Free Dispensary for Sick Children' was opened by Dr. Eustace Smith and Dr. T. C. Kirby at No. 12 Bell Street, Lisson Grove. During the first six years of the Dispensary, which was carried on under the management of a medical committee, relief was afforded to upwards of 20,000 children, and the need of hospital accommodation early became conspicuous." Year by year then the development of the hospital is detailed. From 1881, when Mr. George Hanbury, the present treasurer, first began to devote his time and his purse to the institution, through the gradual appropriation of freehold sites on Paddington Green, in the 'eighties, to 1891 and 1892—those years of dismay at, and final victory over, the infection that somehow contrived to lurk in the existing buildings, in spite of the alteration and demolition of its parts. In 1893 the hospital largely as it exists to-day was planned, and that westernmost side which is chiefly visible to the neighbourhood, and which comprises the board room, secretary's office, and two admirable wards, was then begun. It is to be hoped that the history may be carried further into print, for the interest of the hospital's doings received a fresh importance from the enlargement of this year.

The hospital's committee has hoped for some time to extend the capacities of the out-patient department: there were nearly 16,000 cases last year, and the accommodation was lacking in that differentiation and classified division which, for instance, at Great Ormond Street, have received the latest approval of hospital experts. Before considering the new out-patient department in any detail, it may be well to state a few general facts. The hospital is fortunate in the possession of a square corner site, open at the back, and capable of lateral extension in the future. Simply stated, the new out-patient department will be that improvement to the back of the site which the new wards already mentioned were to the Paddington Green or front aspect of it fifteen years ago. It is true that the square is encroached upon by five houses in Church Street, which will hide the new department from that thoroughfare, but the hospital is the owner of their freehold, and in the future, no doubt, they will have to go. These houses lie, of course, at the north corner of the site.

The King's Fund, to which the hospital owes promises of £2,250 in all, suggested and has approved the revised plans of the new buildings now in progress, and its assistance has enabled the extension to proceed. The total cost is expected to be £11,000, of which £2,800 approximately has yet to be obtained. We may now quote from the report issued last week what is virtually the architect's (Mr. Alan E. Munby's) description of the building. The present out-patients' hall, we learn, is to be retained and enlarged by the addition of the space now occupied by the dispensary and by the consulting rooms. Behind this, or north-east of this, if the position of the compass be preferred, will be an entirely new block consisting of two medical and one surgical consulting rooms, besides receiving, anæsthetic, operating, and recovery rooms, with one more for possible infectious cases. In order that the classification of the out-patients may be carried out smoothly, the sister-in-charge separates them, as they emerge from the entrance lobby, into medical and surgical cases, which are then called to the respective consulting rooms of the two departments. What is now the surgery will be the almoner's room.

Here we may note that a trained almoner will be a new appointment at Paddington Green, but in spite of that, the hitherto existing system of keeping in touch with the homes of the out-patients and of checking possible abuse deserves attention. No subscribers' letters have ever been the means of access to this children's hospital, and the vain attempt to cast what ought to be the hospital's responsibility on to the writer of an annual cheque has never been made here. Intending out-patients have been courteously told that they must bring a card of recommendation from their doctor, who, of course, may or may not be one of the hospital's staff, and this system, recently advocated, we believe, in the North as a novel solution of the difficulty, has always prevented friction between Paddington Green Hospital and the practitioners of the neighbourhood. A more humane, a less mechanical factor in the harmonious working of this system for dealing with the out-patients has been the care and local personal knowledge of the sister, Miss Stubbs, who for four years has had charge of this exacting and unostentatious work. The experienced organisation of which she is still the centre should make the installation of the official almoner in October an easy transition to the new from the old.

When the London County Council approached the hospital with the request that it would treat a certain number of school children, the characteristic reply was made that the institution was always ready to treat as many school children as its extending accommodation allowed, but that further than that no written contract or arrangement could bind it to go! It is also thought, we understand, that the effects of the Insurance Bill, however much modified in committee, can be awaited with equanimity, because the Bill does not appear to contemplate insuring the dependents of the workers, which of course children are, and therefore it would appear that there will still be work for this and other children's hospitals on the familiar lines, to whatever extent other hospitals may be affected by it.

This hospital, which cannot be said to obtrude its claims upon the public in the manner that other, even special, even children or cancer, hospitals are compelled to do, has managed to keep a fairly healthy roll of annual subscribers. Some valuable influence must have been at work to secure this, the most gracious of all methods of support, and its presence is shown also by the vitality of the annual meeting. How few are the hospitals where interest in this yearly function keeps alive. But at Paddington Green there is generally a healthy attendance, which more often than not succeeds in producing a few new annual subscribers. One irrelevant point finally may be added, and that refers to the surprising view than may be obtained from the hospital's leads. The Albert Hall in the far south distance takes on a new dignity, its dome looking like a gigantic bubble through the smoke. In October, it will be remembered, this hospital again opens its doors, when Harlesden and the rest of the adjacent suburbs again will begin to seek admission.

